# The Importance of Partnership in the Rollout of Triple-Drug Therapy to Eliminate Lymphatic Filariasis in the Pacific

**DOI:** 10.4269/ajtmh.21-1085

**Published:** 2022-03-15

**Authors:** Merelesita Rainima-Qaniuci, Hansell Blanche Lepaitai, Rasul Bhagirov, Eswara Padmasiri, Take Naseri, Robert Thomsen, Kimberly Y. Won, Tara A. Brant, Emily Dodd, Motusa Tuileama Nua, Fara Utu, Aifili Tufa, Emi Chutaro, Janet Camacho, Lynette Suiaunoa-Scanlan, Li Jun Thean, Jyotishna Mani, Myra Hardy, Josaia Samuela, Lucia Romani, John Kaldor, Andrew C. Steer, Daniel Faktaufon, Vinaisi Bechu, Flora Naqio, Vine Sosene, Makoto Sekihara, Junko Otaki, Tamara S. Buhagiar, Aya Yajima

**Affiliations:** ^1^ World Health Organization, Suva, Fiji;; ^2^ World Health Organization, Apia, Western Samoa;; ^3^ World Health Organization, Geneva, Switzerland;; ^4^ Samoa Ministry of Health, Apia, Samoa;; ^5^ U.S. Centers for Disease Control and Prevention, Atlanta, Georgia;; ^6^ American Samoa Department of Health, Pago Pago, American Samoa;; ^7^ Pacific Island Health Officers’ Association, Honolulu, Hawaii;; ^8^ Pacific Island Health Officers’ Association, Pago Pago, American Samoa;; ^9^ Murdoch Children’s Research Institute, Melbourne, Australia;; ^10^ Kirby Institute in the University of New South Wales, Sydney, Australia;; ^11^ Fiji Ministry of Health and Medical Services, Suva, Fiji;; ^12^ Tuvalu Ministry of Health, Fakaifou, Tuvalu;; ^13^ Japan International Cooperation Agency, Tokyo, Japan;; ^14^ Australian Institute of Tropical Health & Medicine at James Cook University, Smithfield, Australia;; ^15^ World Health Organization, Manila, Philippines;

## Abstract

We discuss the experience of some Pacific island countries in introducing the new WHO-recommended treatment protocol for lymphatic filariasis—a triple-drug therapy composed of ivermectin, diethylcarbamazine, and albendazole. The successful rollout of the new treatment protocol was dependent on strong partnerships among these countries’ ministries of health, WHO, and other stakeholders. Effective communication among these partners allowed for lessons learned to cross borders and have a positive impact on the experiences of other countries. We also describe various challenges confronted during this process and the ways these countries overcame them.

## INTRODUCTION

The Pacific Ocean is the largest of the world’s oceans, covering one third of Earth’s surface and containing about 3,000 islands in 22 countries and territories. ^1^ Geographically, these islands are spread in a vast ocean, and each island is home to distinct cultures, languages, and political systems. Pacific Islanders speak more than 1,000 indigenous languages, in addition to French in former and current French territories, and English in most of the countries and territories. The climate also varies throughout the Pacific islands, depending mainly on latitude. Nevertheless, there are some cultural commonalities, and all Pacific Islanders share profound respect for traditional community values. Pacific Islanders also frequently travel between islands and maintain close intercultural communication.

Lymphatic filariasis (LF), commonly known as elephantiasis, is a disease caused by filarial worms that live in the lymphatic system and commonly leads to chronic manifestations such as lymphedema, elephantiasis, and hydrocele. LF has been a scourge in many of the Pacific islands for centuries, with rates among the highest in the world. The first record of LF elephantiasis in the Pacific was made by Captain James Cook in Tonga while traveling in 1785. ^1^ Studies carried out in the 19th century by V. Gunson Thorpe and Patrick Manson found microfilariae in blood films in Fiji, Tonga, and Samoa. ^2^ Efforts to control the “elephantoid” diseases, as they were termed by this duo in 1896, have a long history in the Pacific, starting with national community vector control in Fiji in 1944, then moving to the Cook Islands, Samoa, and Tahiti. ^1^ The strategies included mass cleanup campaigns and the use of insecticides such as dichlorodiphenyltrichloroethane for indoor residual spraying. Treatment of LF using various medicines, such as antimonials, arsenoxides, cyanine dyes, and piperazine derivatives, was also carried out, but with little success until the introduction of diethylcarbamazine (DEC) in 1947. ^1^ Mass drug administration (MDA) in the Pacific using DEC alone began in the early 1950s through 1960s in American Samoa, Fiji, French Polynesia, Niue, Palau, Samoa, the Cook Islands, Tokelau, Tonga, Tuvalu, Wallis, and Futuna. ^1^ Multiple rounds of MDA using DEC with high population coverage was found to be greatly effective in reducing the prevalence of infection in general. However, the overall impacts of such MDAs varied by country or territory as a result of nonstandardized implementation of MDA modality and duration.

Following the endorsement of World Health Assembly resolution WHA50.29 in 1997, the Pacific Program for the Elimination of Lymphatic Filariasis (PacELF) was launched in 1999 as an alliance of 22 Pacific island countries and territories, donors, and partner agencies, 1 year prior to the launch of the Global Program to Eliminate Lymphatic Filariasis. ^3^ The WHO Representative Office in the South Pacific, located in Fiji, has long served as a secretariat of PacELF in managing and facilitating information sharing among the Pacific island countries and territories, and as a network of support from donor and partner agencies to Pacific island countries and territories. ^1^

In 1999, Samoa was the first country to roll out MDA using a combination of DEC and albendazole as the PacELF strategy. ^4^ By the end of 2001, 11 more countries and territories also rolled out their MDA campaigns successfully using this double-drug therapy. Annual rounds of MDA have continued throughout the Pacific, and by the year 2020, 8 of 16 LF-endemic countries and territories (50%)—namely, Kiribati, Niue, Palau, Republic of the Marshall Islands, the Cook Islands, Tonga, Wallis and Futuna, and Vanuatu—had achieved elimination of LF as a public health problem and received validation of that status by the WHO. ^5^

## LYMPHATIC FILARIASIS SITUATION IN THE PACIFIC BEFORE THE INTRODUCTION OF TRIPLE-DRUG THERAPY MDA

Some of the remaining Pacific island countries and territories, including American Samoa, Fiji, French Polynesia, New Caledonia, Samoa, and Tuvalu, continue to face challenges in achieving LF elimination targets as a result of persistent transmission or recrudescence of transmission. The prevalence of LF in these countries and territories was historically as high as 40% in the 1950s, and all regions have highly efficient and sub-periodic diurnal *Aedes* mosquitoes as the principal vectors of *Wuchereria bancrofti*. ^1^^,^ ^5^ All these countries also have a long history of MDA for LF, resulting in implementation fatigue, which in turn hindered the achievement of consecutive rounds of high-coverage MDA. In addition, the target threshold for stopping MDA in areas where *Aedes* is the primary vector is less than that in areas where *Anopheles* or *Culex* is the main vector because *Aedes* species are known to be more efficient transmitters of the filarial parasite. ^6^ As a result, in recent years, the above mentioned countries have all failed a transmission assessment survey (TAS) or pre-TAS—either countrywide, in specific implementation units, or in some hotspots within endemic implementation units. ^5^^,^ ^7^ A series of studies has also demonstrated persistent transmission of LF in some of these countries, and research partners have provided continuous feedback to relevant health ministries/departments to emphasize the critical importance of intensifying and sustaining LF interventions to relevant health ministries/departments. ^8^^–^ ^16^ All these factors combined have made the development of a new strategy to accelerate LF elimination crucial.

In November 2017, the WHO issued a new guideline recommending co-administration of ivermectin, DEC, and albendazole (IDA) as a triple-drug therapy to accelerate the elimination of LF. ^17^ This recommendation followed a large, multicenter community study that demonstrated the superiority and safety of IDA compared with conventional double-drug combination for clearing larval filarial parasites from the blood of infected persons. ^18^^–^ ^20^ IDA was regarded as not only a game-changer to reduce the timeline to eliminate LF, but also an opportunity to revive LF elimination efforts in the remaining LF-endemic countries and territories in the Pacific. ^5^ In parallel with the publication of the new guideline, WHO headquarters, the WHO Regional Office for the Western Pacific (WPRO), and WHO country offices began to discuss with countries and key partners a plan to facilitate rollout of IDA in the Pacific.

## KEY FACTORS IN THE SUCCESSFUL ROLLOUT OF TRIPLE-DRUG THERAPY MDA IN THE PACIFIC

**Figure 1.**
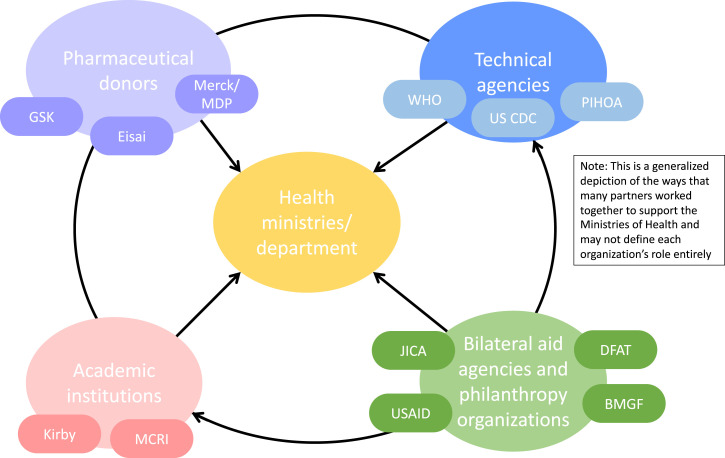
A depiction of the many partners from various institutions involved in supporting the ministries/departments of health in ivermectin, diethylcarbamazine, and albendazole rollout in the Pacific. BMGF = Bill & Melinda Gates Foundation; DFAT = Department of Foreign Affairs and Trade, Australia; JICA = Japan International Cooperation Agency; MCRI = Murdoch Children’s Research Institute; PIHOA = Pacific Island Health Officers Association; USAID = U.S. Agency for International Development.

Given the limited health systems and human resource capacity in many of the Pacific island countries and territories, the rollout of IDA in the Pacific was facilitated through a strategic partnership of international agencies and research partners with relevant health ministries through technical, operational, and financial support in building tools, sharing knowledge, and adapting experiences and lessons learned from one country to another. A depiction of the many partners involved in supporting the ministries/departments of health in the rollout of IDA in the Pacific can been seen in Figure 1. For instance, IDA implementation was supported by the WHO and the Japan International Cooperation Agency (JICA) in Samoa and Tuvalu, and by the U.S. CDC and the Pacific Island Health Officers Association (PIHOA) in American Samoa. The rollout of IDA in Fiji started through a strategic collaboration between the Ministry of Health and Medical Services and the Australian partners, the Murdoch Children’s Research Institute (MCRI) and the Kirby Institute at the University of New South Wales, to integrate IDA with an ongoing scabies control project testing the safety and efficacy of ivermectin-based MDA. Although pre- and post-IDA MDA monitoring and evaluation was also supported extensively by various academic and research partners, these aspects of the rollout are discussed elsewhere and this article focuses on programmatic implementation of IDA.

The remainder of this section summarizes the eight key factors supporting the successful rollout of IDA in the Pacific through strategic partnership: 1) facilitating country dialogue and reviving the political commitment of the government, 2) facilitating the sharing of experiences and lessons learned across countries, 3) refining treatment dose from age-based to weight- or height-based dosing, 4) developing a detailed and comprehensive micro-plan with community profiling, 5) strengthening the health workforce’s capacity on mass campaigns and pharmacovigilance, 6) enhancing stakeholder and community participation through advocacy and social mobilization, 7) using a mixed distribution strategy with directly observed treatment to achieve high coverage, and 8) enhancing the collection and strategic use of data on MDA coverage .

### Key factor 1: Facilitating country dialogue and political commitment.

The introduction of IDA was leveraged as a valuable opportunity to revive the commitment of national governments to eliminate LF. For instance, unlike many of its neighboring countries in the Pacific, American Samoa was not actively conducting any LF elimination activities when IDA was introduced. As such, planning for the transition to IDA required a rebuilding of the LF program infrastructure, which had not been fully active since 2007. To engage all sectors of the government fully, key leaders from the American Samoa Department of Health (ASDOH), PIHOA, and the U.S. CDC met with the governor and lieutenant governor of American Samoa to present the LF situation in the territory and to request an official declaration of LF as a public health threat. Once issued, the proclamation from the executive office was the linchpin in the activation of LF-specific operations.

The WHO and the U.S. CDC facilitated discussions between Samoa and American Samoa to explore the possibility of introducing IDA simultaneously, and thereby coordinating and synchronizing their MDA for better impact. For this purpose, a binational meeting, organized by both governments’ ministry or department of health and facilitated by the WHO and the U.S. CDC, was held from November 29 to December 1, 2017, in Pago Pago, American Samoa. The purpose of the meeting was to review jointly the progress and challenges in eliminating LF in American Samoa and Samoa, plan coordinated activities to respond to the recent resurgence of LF, and stop transmission of the infection in both areas. During the meeting, the governments of both Samoa and American Samoa agreed that, given the patterns of intra- and inter-island migration, coordination of planned MDA activities was needed to maximize access to treatment, coverage, and impact. Both islands renewed their commitment to a joint effort to eliminate LF. After the meetings, in the first quarter of 2018, both governments developed new LF elimination action plans that clearly outlined the activities required to implement MDA with high coverage and to monitor its impact.

### Key factor 2: Facilitating the sharing of experiences and lessons learned.

Facilitating the sharing of experiences and lessons learned from first adopters across the Pacific island countries and territories was an important factor for success. In February 2018, the WPRO organized the program managers meeting on neglected tropical diseases (NTDs) in the Pacific in Nadi, Fiji. ^21^ The meeting included representatives of 17 Pacific island countries and territories as well as partner agencies. The new WHO guidance on IDA was introduced to the meeting participants, and Samoa shared its plan to roll out this new strategy, encouraging other countries to consider its adoption. MCRI and the Kirby Institute also participated and presented updates from their project to demonstrate the effectiveness and safety of MDA using ivermectin for the public health control of scabies in Fiji and the Solomon Islands, indicating opportunities for integrated control of LF and scabies.

In August 2019, the WHO organized a meeting in Bangkok, Thailand, for global review of the initial use of IDA and for planning the accelerated elimination of LF. Representatives of LF elimination teams in Fiji, Samoa, and American Samoa who already had experience implementing the new MDA regimen shared their practical experience, challenges, and feedback in implementing IDA. Other countries that were eligible and preparing for its adoption such as French Polynesia and New Caledonia also participated, and representatives discussed their plan and concerns in coordinating with partners, mobilizing resources, and preparing and implementing MDA using IDA.

In September 2020, the WPRO again organized a program managers meeting on NTDs in the western Pacific. ^7^ The meeting included health ministry representatives from 18 countries in the region. The meeting provided an opportunity for those in key countries and territories to share their challenges and the importance of MDA implementation for various NTDs, including the prevention and management of severe adverse events and the use of mobile technology in IDA rollout.

### Key factor 3: Refining treatment dosing strategies.

American Samoa, Fiji, Samoa, and Tuvalu historically used age-based dosing instead of weight- or height-based dosing of albendazole and DEC for LF elimination. The introduction of IDA in Fiji started as part of a large multicenter community trial to compare the safety and efficacy of triple-drug (IDA) to double-drug treatments in Rotuma and the Gau islands of the eastern division in 2017. ^20^^,^ ^21^ The large data set from this multicenter community trial, which included age, height, and weight, was used to develop a practical height-based dosing algorithm to decrease the frequency of underdosing that can occur with age-based dosing, which was the standard practice in the Pacific, and to avoid the logistical difficulties of weighing individuals in remote, resource-limited settings where working scales are not common. ^22^ The national LF elimination program in Fiji adopted height-based dose poles in its programmatic triple-drug therapy MDA rollout in the northern division in 2019. The dose poles were made by local school and college students, which also facilitated community engagement. Following the experience of Fiji, Tuvalu also adopted the height-based dosing approach for IDA rollout. The WHO and JICA supported the production of dose poles in Fiji and shipped them to Tuvalu, along with other supplies procured outside Tuvalu, for MDA implementation.

By contract, American Samoa and Samoa decided to shift from age-based dosing to weight-based dosing to minimize underdosing while ensuring safety, considering their typically greater body mass index than in other parts of the world. The WHO supported recalculation of the weight-based dosing table using the height–weight–age data of the Samoan population, which was presented to and endorsed by the Mectizan Expert Committee in July 2018. Because both countries had limited access to available weight scales, JICA, for Samoa, and the U.S. CDC and PIHOA, for American Samoa, supported the procurement and import of scales.

During the MDA rollout, MDA teams noticed scale malfunctioning and discovered the scales needed to be hardy enough to withstand heavy individual weights if the weight-based dosing was to be adopted. In addition, in American Samoa, it was sometimes difficult to obtain accurate readings if a flat surface was not available for the scale. The MDA teams overcame this challenge by carrying pieces of plywood to create suitable surfaces.

### Key factor 4: Developing a detailed and comprehensive micro-plan with community profiling.

In all countries and territories, careful micro-planning was a key driver to successful MDA. Micro-planning started with a line listing of health zones and villages, with estimated populations and existing schools in each. MDA supervisors and team members were then assigned to each health zone and village. The required resources, including medicines, other supplies, volunteers, and vehicles, were identified, and the cost to procure or arrange such resources was estimated to ensure the allocation of sufficient resources.

In all the countries and territories, a series of consultations was held with various stakeholders, such as national and local policymakers, community leaders, religious leaders, school principals, and both public and private health facilities and clinicians. These consultations provided an orientation to the MDA campaign and sought stakeholder cooperation and support. In Fiji, collaboration with other government bodies such as the Fiji Bureau of Statistics, the Ministry of Education, and the Ministry of iTaukei Affairs enhanced the effectiveness of MDA distribution. For example, the Ministry of Education facilitated the acquisition of third-party consent from parents prior to the MDA, enabling distribution to children at schools. The Fiji Bureau of Statistics shared information regarding population size at the community level as well as geographic demarcations. The Ministry of iTaukei Affairs provided a means for disseminating information about the program from provincial councils to the local level, enabling access to traditional Fijian villages.

In American Samoa, to facilitate program implementation, ASDOH decided to use an existing United Health Command structure, analogous to a public health emergency response system. The United Health Command was comprised of various governmental departments and agencies, including the Public Health Emergency Preparedness Program. A United Health Command structure is often activated within ASDOH when health interventions need to be prioritized, because it allows agencies with different functional authorities and responsibilities to work together. This type of system had recently been used to address Zika in 2016. LF-specific operations groups were formed under the United Health Command to oversee MDA program activities, including data management, logistics, training, communications, and drug administration.

Consultations with sub-national authorities provided an opportunity to refine the micro-plans. For example, through consultations of the Ministry of Health with public and private health practitioners in Samoa, some of the practitioners agreed to be designated as on-call doctors during MDA campaigns to lead case investigation and risk communication in case of any severe adverse events. In American Samoa, informational meetings with the Office of Samoan Affairs, comprised of Pulenu’u (village mayors), were important in planning MDA timing and visits in each village. In Tuvalu, consultations involved Kaupule and Falekaupule (island councils), which supported community group consultation in each island setting with faith-based groups such as youth groups, church choruses, and university student groups.

### Key factor 5: Strengthening health workforce capacity.

The introduction of IDA provided an opportunity to refresh the knowledge and motivation of the health workforce involved in LF elimination activities and to strengthen even further their capacity on the pharmacovigilance associated with MDA. All four countries and territories had a long history of the fight against LF and have been implementing MDA campaigns since 1999 or 2000. Programmatic fatigue from long-running annual MDA programs during the past two decades has been observed increasingly among the staff in the health ministries and also among community drug distributors. Regular training of those in the health workforce was no longer implemented regularly and systematically. However, because this was the first time a new medicine (ivermectin) had been added, and the number of tablets to be ingested by individual community members increased significantly, it was essential to retrain all health staff involved in MDA campaigns.

In all the countries and territories, the ministries of health and partner agencies facilitated large-scale training sessions for those in the health workforce who are involved in the nationwide MDA campaign. There were question-and-answer sessions throughout the training, and significant time was spent to ensure all questions were responded to properly by the facilitators. There was also a role-play of drug administration, including how to weigh people using a scale, as many of the participants in the training sessions had no experience in using a scale to weigh either themselves or others. The training provided them with the assurance that this new combination of medicines was safe and would be effective in finally eliminating LF. It also helped to improve the workers’ preparedness to monitor, manage, and report any severe adverse events associated with other MDA campaigns.

As mentioned earlier, by the time Samoa implemented its first MDA using IDA in 2018, Fiji had already adopted IDA via community trials in 2017. Communication materials developed by the Fiji team for their trials were shared with Samoa and used as references to develop a new set of training modules for Samoa’s health workforce. Samoa, in turn, shared the translated materials with American Samoa. The training emphasized the roles and effectiveness of the three medicines in LF elimination, the importance of and key factors in achieving high treatment coverage, and the prevention and management of adverse events with the support of a pharmacovigilance team from the WHO WPRO.

Because American Samoa is a territory of the United States, regulatory requirements unique to the United States had to be addressed prior to the launch of triple-drug therapy MDA to ensure its safety. Although the WHO had approved a supply of medicines for American Samoa, the medicines had not been cleared for widespread use in the United States or its territories. DEC was not approved by the U.S. Food and Drug Administration (FDA) for commercial distribution; ivermectin and albendazole were approved only for prescription use. Therefore, the ASDOH had to seek U.S. FDA approval to use these medicines for MDA. The request to the U.S. FDA for expanded access to the medicines involved a lengthy application that included detailed information about the safety profiles of the medicines, manufacturing information, protocols on how the MDA would be conducted, and a detailed pharmacovigilance plan. Because this expanded-access mechanism was an exception to official regulatory requirements, the ASDOH was required to obtain written informed consent or parental permission from every person participating in MDA, in comparison to the verbal consent approach generally used in MDA campaigns.

### Key factor 6: Enhancing stakeholder and community participation.

In all four countries and territories, a high priority was given to social mobilization activities aiming to reinforce consistent messaging in the local language about the targeted diseases and the safety of MDA. This was necessary to build trust within the communities and was a cornerstone of achieving effective coverage.

The communication and social mobilization plans had three components: 1) mass media campaigns, 2) consultations with local stakeholders, and 3) community social mobilization sessions. Branding and messaging were usually done in collaboration with the communications teams of the health ministries at a national level. Simplified messages were developed to inform communities, and consistent messaging targeting individual behavior change was delivered. It made an impact because targeted populations were able to understand the cause and treatment of scabies, LF, and soil-transmitted helminths. It aimed to ensure that communities understood the rationale for reducing disease prevalence, and were assured of MDA safety by trusted and credible top health officials through media and other communication channels. This was particularly important in Samoa, where there was already heightened public attention focused on the health system because of the adverse events that followed an immunization campaign that had occurred a few weeks before the MDA campaign. Community members particularly appreciated information on the enhanced adverse event monitoring protocols and pathways after treatment.

The mass media campaign included press releases, TV and radio discussions, newspaper articles, and messaging through churches. It targeted internal and external media, health-care services, and in-country stakeholders such as provincial offices, district offices, schools, local businesses, faith-based organizations, village leaders, women’s groups, youth groups, and selected private bodies and civil society organizations.

In all countries and territories, community health workers, village leaders, and village members with local knowledge of the location of households and where people were living were recruited to support MDA teams. Volunteers often included members of local nongovernmental organizations such as the Red Cross. The recruitment of individuals who were highly trusted by community members greatly helped the MDA teams facilitate drug distribution and played a vital role in increasing MDA compliance in the community. For example, the streets in Pacific island countries typically do not have names or household numbers, and there are no village registers that enumerate the cornerstone households or persons available to the teams in advance of the MDA. Therefore, the recruited, trusted local community members were essential in confirming an accurate village census, checking whether anyone was missed, and verifying coverage against a known denominator.

To maintain high motivation and visibility of MDA teams, uniform T-shirts, hats, and bags were produced and procured with the support of partners, and were greatly appreciated by the teams and community alike. Adequate compensation for hours worked and timely remuneration of overtime for drug distributors and volunteers—including nurses and non-health professionals such as general workers and drivers—were also found to be major motivators for maintaining high efficiency and commitment from the MDA teams.

The communication campaigns escalated in terms of visibility, frequency, and information detail as the MDA campaigns approached. In Samoa, a launch ceremony was organized on the first day of the MDA campaign, with the prime minister and cabinet members taking MDA medicines in public to encourage citizen participation. Similarly, in American Samoa, key leaders from the ASDOH and other top government officials, as well as CDC and PIHOA staff, helped launch the campaign by receiving the three medicines in public at a televised event.

### Key factor 7: Using a mixed distribution strategy with directly observed treatment.

All countries and territories used multiple drug distribution strategies to maximize the coverage through 1) school visits, 2) house-to-house visits, and 3) fixed sites, with some variations to reach as many unreached populations as possible.

In Samoa, house-to-house visits for MDA were preceded by MDA in schools. The Ministry of Education, Sports, and Culture and school principals in both public and private schools gave their fullest cooperation for MDA. Written consent was obtained from parents to administer MDA at school. Preschools and primary and secondary schools were visited by MDA teams with at least one nurse per team. Many schoolteachers and principals helped enthusiastically with the MDA activity, and some even organized refreshments for the MDA teams. Some parents also went to preschools and primary schools to see their children taking the medication. In Samoa, starting the MDA in schools was critical to achieving high coverage, because through preschools, primary schools, and secondary schools, nearly 40% of the total population could be reached. Marking of recipients’ finger using indelible ink only after directory observed treatment ensured avoidance of duplicate treatment at schools and house-to-house visits.

Initially, in American Samoa, fixed posts such as places of business, churches, schools, and clinics were used as distribution sites. However, the ASDOH soon realized updates at some sites were much less than anticipated and certain populations were not being reached, and there was a rapid shift to a mixed distribution strategy. Some fixed posts were maintained, whereas less-productive sites were closed and replaced with house-to-house distribution. This prompt shift was possible because an electronic data collection system allowed real-time monitoring of coverage by site and village. In addition, each MDA team in American Samoa included a registered clinician to supervise distribution and observe each participant taking the medicines.

### Key factor 8: Enhancing the collection and strategic use of data.

For the first adopters of IDA, various data reporting tools such as the MDA registration form, tally sheets, and serious adverse events reporting forms had to be newly developed, and training on their use was needed. Fiji, Samoa, and Tuvalu used paper-based data reporting and monitoring, whereas American Samoa used a mobile health system.

In all three countries using a paper-based system, coverage data were entered manually by the community drug distributors onsite in hard-copy registration books and tally sheets, as they treated people. All hard-copy registration books were then sent to the central data team, which extracted data from the registration books and entered them into an Excel database. In the field, the team coordinator checked the registration books on a daily and regular basis for accuracy, completeness, and any follow-up required. At the fixed center, the team coordinators held discussions with the community drug distributors every morning about the progress of the MDA, issues identified, and solutions. These included monitoring adverse events and referring individuals to the medical officers who were deployed in the field. This daily monitoring and feedback enhanced the engagement and motivation of MDA teams in working toward high coverage.

In Tuvalu, the shipping services scheduled for outer islands were limited, and therefore sending data on paper forms to the Ministry of Health was sometimes delayed by 3 months or more. Although Internet service was not reliably accessible because of a lack of connectivity and high costs, alternative communications via traditional phone calls were used to relay the summarized results.

In American Samoa, the ASDOH worked with the U.S. CDC and PIHOA to develop an electronic data collection system. Electronic tablets were programmed with an Epi Info version 7 (U.S. CDC) questionnaire and were used to collect participants’ basic demographic information, including age, gender, and village of residence. In addition, the tablets were programmed to assist with correct dosing of the medicines. Upon entering a participant’s weight, the correct number of pills of each of the three drugs was displayed on the screen. All relevant health department personnel were trained on how to use the tablets and enter data. This innovation enabled immediate identification of groups, and areas with low treatment coverage during daily debrief meetings, and allowed the ASDOH to adapt the MDA strategy to target these groups and conduct mop-up in the areas with low coverage.

The WHO encourages all countries and territories to implement coverage surveys. The Supervisor’s Coverage Tool, a WHO-recommended rapid-coverage assessment tool that uses lot quality assurance sampling to monitor MDA coverage in a given supervision area, was used, when possible, to identify areas in need of mop-up. ^23^ In American Samoa, for instance, the reported coverage of the first IDA round was approximately 55%, based on the 2010 census data. However, an independent coverage evaluation was conducted 3 months after the MDA and indicated a coverage of approximately 73%, exceeding the WHO recommended target of 65%. The large discrepancy between the two coverage estimates was likely a result of inaccurate census data and is a challenge that has been observed previously. ^24^ This experience highlights the importance of up-to-date and accurate census data and, if not possible, validating reported coverage estimates. However, it is also notable that implementation of a coverage survey was often unfeasible in countries and areas where human resources and funding for MDA are extremely limited, and where options for travel arrangement to multiple outer islands are limited, as in the case of Tuvalu.

## TURNING CHALLENGES INTO OPPORTUNITIES TO REACH THE UNREACHED AND STRENGTHEN HEALTH SYSTEMS IN THE PACIFIC

Successful rollout of IDA in the Pacific was possible as a result of existing structures and capacity at the national level to plan and implement LF elimination activities, along with the support and partnerships from the PacELF network. American Samoa was an exception; because they had stopped MDA 10 years prior, they had to plan and prepare for the MDA campaign with limited institutional memory and thus required more operational support, with the U.S. CDC and PIHOA adopting this role.

When operational capacity and partner support were in place, tools to operationalize MDA campaigns were needed. For the first countries and territories to implement an IDA strategy, a series of tools was developed through partnerships between health ministries and partner agencies. These partnerships supported every step of MDA preparation and implementation in the specific context of the Pacific. This process also offered an opportunity to review the conventional approaches used up to that point, improve each component of MDA preparation and implementation, and increase the health systems’ capacity to enhance the effectiveness, efficiency, and safety of MDA. Close coordination and communication between stakeholders and the WHO enabled the identification of any technical, operational, or financial gaps and the mobilization of necessary resources in a timely manner.

Despite these strengths and partnerships in place, the rollout of IDA in the Pacific island countries and territories had various unique challenges characterized by their small populations in remote island settings.

First, small Pacific island countries and territories have a limited number of health professionals. In the health ministries, even at the central level, a small number of staff is usually responsible for multiple administrative functions. MDA campaigns for LF require all levels of the limited health workforce, including primary health-care workers and community volunteers, to focus on the organization and delivery of drug administration nationwide within a short amount of time. When other competing public health priorities occur, the entire health workforce must be deployed and MDA campaigns have to be postponed.

For example, a measles outbreak started in Samoa in September 2019, the month the second IDA campaign was originally scheduled. In the end, the outbreak included more than 5,000 cases and 80 deaths out of the population of approximately 200,000. ^25^ This led the entire health ministry to focus on a measles, mumps, and rubella vaccination campaign, postponing the second round of the IDA campaign. Subsequently, in March 2020, the WHO declared the COVID-19 outbreak a global pandemic. Both Samoa and American Samoa declared a state of emergency that same month, and enforced border closures and strict quarantine measures, including a travel ban between Samoa and American Samoa. So far, this strategy has been successful, but it has come at the expense of health ministry and/or department focusing solely on the COVID-19 pandemic response. American Samoa managed to implement an impact assessment after the second round of IDA in September to October 2020 on a smaller scale than the survey after the initial round of IDA. However, the next rounds of IDA in both Samoa and American Samoa have been postponed further because of the launch of the COVID-19 vaccine rollout in 2021. In Samoa, this means the second round of IDA remains postponed. In American Samoa, the ASDOH implemented MDA from October 2021-January 2022, which represented a two year gap between the second and third rounds of IDA.

Second, small Pacific islands have logistic and infrastructure challenges including limited telecommunications, infrequent transport links, poor infrastructure, and high operational costs. Procurement of various goods and supplies, such as dose poles, weight scales, and indelible ink markers, need to be imported from other countries. Large-scale and high-quality printing of a variety of materials, including training materials, posters, leaflets, banners, booklets for drug distributors, and reporting and tally sheets, is often not available, and bulk printing needs to be done and shipped from other countries. Rental of limited vehicles and boats for an extended duration as well as traveling to every single outer island all pose unique logistical and financial challenges. Finding storage space for large amounts of medicines and other supplies, particularly those needing temperature control, is also a challenge. Since the COVID-19 pandemic started in early 2020, commercial flights to and from Tuvalu have been suspended, except for several small repatriation flights. As a result, the shipment of medicines from the pharmaceutical donors needed for the second round of IDA was interrupted for nearly a year. To address this challenge, the WHO country office for Fiji had to step in to receive the medicines for Tuvalu, and then forward them using a local cargo ship, which required extra resources and time. The medicines finally arrived in Tuvalu in April 2021, 10 months late.

Therefore, strong partnerships across countries and territories and among health ministries, developmental donors, and partners are essential to achieving LF elimination in the Pacific. Also, because MDA is a significant undertaking, both cost-effectiveness and opportunities to leverage MDA platforms to integrate delivery of other public health services and to maximize the reach to previously unreached populations should be explored. The support from the MCRI and the Kirby Institute to facilitate integration of the public health control of scabies in the IDA rollout is an innovative example that can serve as a model for many other countries where LF and scabies are co-endemic. ^22^^,^ ^26^ Importantly, this model is being taken forward by the World Scabies Program in both Fiji and the Solomon Islands. ^27^ The decision to use the United Health Command structure in American Samoa also allowed the ASDOH to adapt an existing system quickly and apply it to LF activities. After the successful introduction of IDA, the ASDOH is now poised to incorporate lessons learned from MDA to continue strengthening their emergency response system. Because the ASDOH is responsible for all health-related interventions in American Samoa, opportunities to strengthen its health systems are critical to building and improving efficiency in a setting where resources are limited. Moving forward, lessons learned from the LF MDA campaigns will be instrumental in improving the health system for future interventions.

It is important to recognize that investment in LF elimination activities contributes to strengthening overall health systems. Because LF MDA is one of a few platforms to deliver health interventions and services to all age groups in an entire target area, whether provincewide or nationwide, it provides a valuable opportunity to strengthen the underlying health systems to enable such service delivery to reach all the population in need, even hard-to-reach populations, more safely and efficiently. For instance, ensuring the safety of MDA and preventing, managing, and reporting any associated adverse and severe adverse events is one of the most essential components in preparing and implementing MDA. This reinforces collaboration between NTD programs and pharmacovigilance teams in the health ministries on issues such as safety surveillance, causality assessment, and risk communication. Cascade training of the health workforce on MDA preparation and implementation strengthens health literacy and builds logistics capacity to organize community outreach, data management capacity to report and monitor progress, and technical capacity to prevent, manage, and report adverse events. MDA can strengthen health information systems by providing an opportunity to review and improve data reporting pathways and tools, and thereby validate functioning of the link between health facilities and national health information systems. American Samoa demonstrated an example of the introduction of mobile technology for its health emergency operation using MDA as an entry point.

## THE WAY FORWARD FOR LF ELIMINATION IN THE PACIFIC

As the rollout of COVID-19 vaccines progresses, all four countries and territories discussed here have been planning to continue or resume their delayed LF MDA campaigns in 2021 or 2022. In addition, the Pacific territories of French Polynesia and New Caledonia plan to introduce IDA in 2022 in areas where LF transmission persists.

The COVID-19 pandemic has called for people to acquire health information and adopt behaviors such as practicing social distancing, wearing face masks, and washing hands as the most effective non-medical interventions. This has highlighted the importance of health literacy and social responsibility to enable people to grasp the reasons behind recommendations and reflect on the outcomes of their actions. ^28^ It has also rapidly given recognition to the significance of community engagement and solidarity for disease control worldwide. We hope this provides a critical boost to enhanced community participation and high coverage of MDA campaigns as well.

However, it is also important to recognize that the impacts of IDA in the Pacific are yet to be assessed fully. Elimination of LF transmission in the Pacific island countries has been challenging historically as a result of the day biting patterns of the dominant *Aedes* vectors. ^5^ Therefore, standard vector control methods, such as bed nets and indoor spraying used in other geographic areas where nocturnal biting mosquitos dominate transmission, are of limited value. The reasons for LF persistence and resurgence in all these countries and territories are unclear; potential explanations include local factors such as a tropical climate and outdoor lifestyle, an abundance of highly efficient day- and night-biting mosquito vectors, and travel and migration within and across islands. The superiority of IDA over the double-drug treatment of microfilarial clearance at 12 months has been reported from trial sites in Haiti, India, and Papua New Guinea. ^19^^,^ ^20^^,^ ^29^^,^ ^30^ Five-year follow-ups of single-dose triple-drug therapy for *W. bancrofti* in Papua, New Guinea, has also demonstrated sustained clearance of microfilariae. ^31^ However, all these countries have nocturnally periodic LF transmitted by *Culex* or *Anopheles* mosquitoes. A large LF community survey in Samoa, conducted 7 to 11 weeks after its first round of IDA in 2018, showed a greater-than-expected antigen prevalence in the study population, and 14 of 18 people diagnosed positive with microfilariae reported taking the IDA pills in 2018, all of which raises concerns about the effectiveness of IDA in Samoa. ^16^ However, in 2019, repeated treatment with direct observation to evaluate the weight-based dosage for these individuals showed that microfilariae were cleared completely by day 7 in 12 of the 13 participants who were monitored, and by day 30 in the remaining participant, which reconfirms the effectiveness of IDA at clearing microfilariae in the short term when taken at the correct dose. ^32^ By contrast, the community trial conducted on Fiji’s Rotuma and Gau islands to compare the efficacy of IDA to double-drug treatment in microfilarial clearance found no difference at 12 months between groups, as well as no added benefit of IDA over double-drug on community microfilarial prevalence at 12 months, despite high reported MDA coverage. ^22^ This apparent short-term microfilarial clearance that is not sustained is in contrast to the Papua New Guinea experience. More work is needed to determine whether this was a result of reinfection, differences in drug levels, or parasite susceptibility to the treatment.

Current guidance from the Global Program to Eliminate Lymphatic Filariasis recommends monitoring LF prevalence among children in sentinel and spot-check sites after only two rounds of IDA. ^17^ The results from Fiji after two rounds of IDA indicate that two rounds may be insufficient to meet pre-TAS targets in the Pacific with *Aedes*-dominant transmission. Because of the logistics of conducting pre-TAS surveys in remote areas, if pre-TAS fails, conducting MDA in the same year is often not possible. Adding the complexities of the COVID-19 pandemic interruptions, proceeding with a third round of IDA may be preferred. In addition, the method of using antigenemia for TAS may no longer be the ideal indicator for determining levels of community transmission because antigen clearance often lags behind microfilarial clearance. The TAS design in settings where IDA is introduced needs to be modified and validated in the field, including in settings with diurnally sub-periodic LF. Currently a number of academic and research partners are coordinating efforts to generate needed scientific evidence through operational research to resolve challenges and determine the next steps to support LF elimination in the Pacific.

The Pacific island nations were innovators in establishing a sub-regional partnership for LF elimination and introducing annual MDA as an LF elimination strategy as early as 1999. Two decades later, the Pacific island nations successfully became the first adopters of the new triple-drug therapy strategy to accelerate LF elimination, and together account for nearly 60% of countries worldwide that have achieved elimination of LF as a public health problem. ^5^ Because of the various challenges unique to isolated small-island settings, innovations such as strategies to reduce the rounds of MDA required to reach the elimination goal are important to lessen the logistical and resource burden for similar countries and territories. Adding to this, the existing strong regional partnership of countries and partner agencies in the Pacific will continue to support adaptation of global strategies to local settings in the Pacific in the most feasible and operational ways possible. This partnership will help accelerate achievement of sub-regional LF elimination by 2030—the new global target date for LF elimination.
